# A Comprehensive Review of Cutaneous Manifestations Associated with COVID-19

**DOI:** 10.1155/2020/1236520

**Published:** 2020-07-05

**Authors:** Hoda Rahimi, Zohreh Tehranchinia

**Affiliations:** Skin Research Center, Shahid Beheshti University of Medical Sciences, Tehran, Iran

## Abstract

The novel coronavirus (SARS-CoV-2), the cause of coronavirus 2019 disease (COVID-19) pandemic, is associated with some cutaneous manifestations. Although the cutaneous presentations of COVID-19 are infrequent, it is of great importance for all clinicians to be aware of these manifestations, as it may contribute to sooner and better diagnosis and management of the disease, even in asymptomatic or paucisymptomatic patients. The reported cutaneous manifestations of COVID-19 are various, dispersed, and sometimes confusing. In this article, all reported cases to date were collected and classified under 6 major groups: maculopapular rash, urticaria, chilblain, vesicular lesions, livedo reticularis, and petechiae. Different characteristics of each group were discussed in detail as well.

## 1. Introduction

On 31 December 2019, a newly emerged pneumonia caused by a novel coronavirus, named SARS-CoV-2, was announced by China [1]. It spread so rapidly until WHO announced coronavirus 2019 disease (COVID-19) as a pandemic condition on March 11. The firstly reported presentations of COVID-19 were like other viral respiratory infections, including high fever and dry cough. However, it might lead to acute respiratory distress syndrome and the mortality rate was quite high [2]. Since then, a wide spectrum of clinical manifestations have been described, ranging from the absence of any symptoms to fever, cough, dyspnea, diarrhea, ageusia, anosmia, and even cutaneous lesions [3, 4]. Although the cutaneous manifestations of COVID-19 are infrequent, it is of great importance for all clinicians to be aware of these presentations, as they may contribute to sooner and better diagnosis and management of the disease, even in asymptomatic or paucisymptomatic patients. This could be a valuable help for epidemiological control of the disease, especially in regions where diagnostic kits are limited [5]. On the other hand, the reported cutaneous manifestations of COVID-19 are various, dispersed, and sometimes confusing. Thus, we aimed to review and summarize the different skin lesions, which have been reported in association with COVID-19 to date, in this article.

## 2. Methods

PubMed and Cochrane were searched with the search terms “skin”, “cutaneous”, and “dermatology”, each in combination with “COVID-19” or “SARS-CoV-2”. All articles including case reports and original articles from the emergence of the disease (31 December 2019) to the submission of the article (9 May 2020) were included except for one article in which all 6 cases had neither positive PCR test nor common symptoms of COVID-19, and the authors presumed that their cutaneous manifestations may be related to SARS-CoV-2 without any documented evidence [6].

## 3. Results

Different cutaneous lesions have been reported in 451 patients with COVID-19 to date (Table 1). However, it is predictable that the cutaneous lesions have been undoubtedly underdiagnosed due to the lack of dermatology consultations in this pandemic situation. The age of patients ranged between 2 months and 89 years. Patients consisted of 244 females and 178 males, while the gender of 29 patients was not reported. Most reported cases were from Spain (379 pts; 84%), followed by Italy (51 pts; 11.3%), France (10 pts; 2.3%), USA (4 pts; 0.9), Thailand (3 pts; 0.7%), Belgium (2 pts; 0.4%), and Kuwait (2 pts; 0.4%). It is obvious that these data are not compatible with the real prevalence of the disease in different countries, since many dermatological cases might not be reported in the literature due to different reasons. Table 1 shows detailed characteristics of reported patients.

Although the cutaneous presentations of COVID-19 are various, they could be categorized in 6 major groups (Tables 1 and 2).

*(1) Maculopapular rash*. Maculopapular rash, with or without pruritus, was the most common cutaneous manifestation of COVID-19, presented in 200 patients (44.4%). The mean age of the patients in this group was 60.4 years (range: 6-89 years). More than half of the patients were female, and the most common localization of the lesions was the trunk. Although 35% of patients were asymptomatic, the most frequent symptom was itching which presented in 57% of cases. This kind of rash is mostly observed in the active phase of the disease.

*(2) Urticaria*. The second most frequent skin rash in COVID-19 patients was urticaria which appeared in 84 patients (18.6%). The mean age of the cases was 47.6 years, while the youngest patient aged 2 months and the eldest 71 years. Again, females were dominant (66%), Spain reported the most number of cases, and the rash appeared mostly within the active phase of the infection.

*(3) Chilblain*. Chilblain occurred in 81 patients (18%) without any history of exposure to cold. This group had some especial characteristics which made it different from the others; it mostly presented in younger patients with a mean age of 31.7 years, and females were involved significantly more than males (68% vs. 32%). Moreover, in contrast to other cutaneous manifestations, which appeared mostly in the active phase, chilblain often presented later in the course of the disease (after the complete recovery, in half of the patients). Furthermore, approximately 10% of the patients were asymptomatic carriers of COVID-19 who had only chilblain and positive PCR test, without any symptom of COVID-19. None of the cutaneous manifestations was reported in asymptomatic carriers, except for chilblain. Its distribution was, as usual, in acral parts. However, the heels and toes were affected significantly more than the fingers (97.5% vs. 2.5%). The most frequent symptom was pain (32.1%).

*(4) Vesicular lesions*. Vesicular lesions, reported in 61 patients (13.5%), usually manifested like chicken pox, consisting of pruritic papulovesicular rashes involving the trunk mostly. However, three patients presented with localized vesicular lesions which was herpetiform [7]. Vesicular rashes were reported only from Italy and Spain, often in the active phase of the disease.

*(5) Livedo reticularis*. Livedo reticularis was reported in 23 patients (5.1%) as generally asymptomatic lesions, affecting both genders almost equally. In two cases, it was unilateral, resolving in few hours without any treatment [8]. They were in contrast with the usual bilateral distribution of the common livedo reticularis which does no subside spontaneously.

*(6) Petechiae*. To date, only 2 cases (0.4%) of petechiae were reported in association with COVID-19 [9, 10], one of which was firstly misdiagnosed and mistreated as dengue [10].

As it may be noticed, cutaneous lesions of COVID-19 may manifest in different forms, in every age and sex, and involve every part of the body. However, the involvement of mucosa has not been reported yet, except for a case of oral herpes simplex virus-1 reactivation in an intubated patient, which seemed to be secondary to the intubation rather than a presentation of the virus [13]. In all groups, pruritus was the most common symptom, except for chilblain which was more associated with pain than pruritus. Generally, the skin lesions were observed in the active phase of infection (61% of cases) and subsided within few days without any treatment. Although the appearance of skin rash in the prodromal phase or asymptomatic carriers was scarce, it is of great importance for all clinicians to keep in mind that cutaneous lesions might be the only symptom of COVID-19, as it would contribute to sooner diagnosis and management of the patients/carriers and better control of the disease spreading. From 451 reported patients, only 296 cases were confirmed by polymerase chain reaction (PCR) test. Due to the lack of the diagnostic kits in this pandemic period, they were performed only for hospitalized or severe cases in most countries. That is why all cases did not have a documented diagnosis and suspected cases were included as well. However, it should be noticed that (1) many of these suspected cases had other typical signs and symptoms of COVID-19; (2) many of them had positive family history of COVID-19 or close contact with infected patients; and (3) almost in all of these cases there was no other explanation for their cutaneous lesions except for COVID-19. Another important point which remained controversial is that whether these cutaneous presentations have any correlation with the severity of the disease or could be used as a prognostic factor. Although Galván Casas et al. reported that chilblain was associated with less severe disease, and livedo reticularis was associated with the most severe forms of the disease [17], the role of confounding factors such as age should be noticed, as well. However, Recalcati reported no correlation between cutaneous presentations and disease severity [6].

## 4. Conclusion

Although these data do not prove that COVID-19 was the definite cause of these skin lesions, they demonstrate that cutaneous lesions should be considered in the spectrum of presentations potentially associated with this infection. In particular, some cutaneous manifestations such as chilblain without any explanation may warn about asymptomatic virus carriers. However, further investigations should be carried out to evaluate the relation between skin lesions and COVID-19.

## Figures and Tables

**Table 1:** Detailed characteristics of COVID-19 patients who developed cutaneous manifestations.

Cutaneous manifestation	Number of patients	Age (years)	Sex	Country	Localization of the skin lesions	Dermatological symptoms	Phase of COVID-19 in which skin lesions appeared	Clinical course of the skin lesions	Taking any medication before the appearance of skin lesions	Method of COVID-19 diagnosis
Maculopapular rash	14 [6]	N/M	N/M	Italy	Mainly trunk	Mild itching or asymptomatic	Active phase	Resolved few days later	N/M	All cases were confirmed by PCR.
	1 [11]	64	F	France	Trunk & flexors	N/M	Active phase	Resolved 5 days later without any treatment	Paracetamol for 4 days (but the cutaneous lesions disappeared despite the drug was not discontinued)	PCR
	1 [12]	59	F	Italy	Trunk & limbs	N/M	Active phase in ICU	Resolved 5 days later without any treatment	N/M	PCR
	1 [12]	89	F	Italy	Trunk & limbs	N/M	Active phase in ICU	Resolved 8 days later without any treatment	N/M	PCR
	1 [12]	57	M	Italy	Generalized	Itching	Prodromal phase	Reduced 10 days later, after taking levofloxacin and hydroxychloroquine	N/M	PCR
	2 [13]	N/M	N/M	France	Face & upper body	Itching	Active phase	Resolved a few days later	N/M	Both cases were confirmed by PCR.
	1 [14]	58	M	USA	Trunk & extremities	Itching	Active phase	Began to improve 3 days later without any treatment	Azithromycin+benzonatate	PCR
	1 [15]	20	M	USA	Generalized, sparing the face	N/M	Active phase	N/M	N/M	PCR
	1 [16]	6	M	Thailand	Generalized	Itching	Active phase	Resolved 5 days later without any treatment	No medication	PCR
	1 [9]	57	F	France	Trunk & limbs	Asymptomatic except for burning sensation of palms	Active phase	Resolved 9 days later without any treatment	Paracetamol	PCR
	176 [17]	Mean age: 55.3	F: 98 M: 78	Spain	N/M	Asymptomatic: 64 Pain: 4 Burning: 9 Itching: 99	Prodromal phase: 8 Active phase: 108 Convalescence phase: 60	Resolved few days later (mean: 8.6 days)	N/M	122 cases were confirmed by PCR. Others were not tested.
Urticaria	3 [6]	N/M	N/M	Italy	Mainly trunk	Mild itching or asymptomatic	Active phase	Resolved few days later	N/M	PCR
	1 [18]	32	F	Spain	Generalized	Itching	Active phase	Resolved 5 days later after taking antihistamines	Azithromycin+hydroquinone	N/M
	1 [19]	27	F	France	Face & acral parts	Itching	Prodromal phase	Slow improvement after taking antihistamines	No medication	PCR
	2 [6]	N/M	N/M	France	Face & upper body	Itching	Active phase: 1 Prodromal phase: 1	Resolved a few days later	N/M	Both cases were confirmed by PCR.
	1 [20]	71	M	Belgium	Generalized	Asymptomatic	Active phase	Resolved a few days later after taking bilastine	No new medication	All cases were confirmed by PCR.
	1 [20]	39	F	Belgium	Generalized	Itching	Active phase	Resolved a few days later after taking bilastine	No medication	N/M
	1 [16]	2 months	F	Thailand	Generalized except for palms & soles	Itching	Active phase	Resolved 9 days later without any treatment	No medication	PCR
	1 [21]	37	F	Italy	Trunk, neck & face	Asymptomatic	Active phase (10^th^ postpartum day)	Resolved 8 days later without any treatment	Acetaminophen	N/M
	73 [17]	Mean age: 48.7	F: 47 M: 26	Spain	N/M	Asymptomatic: 4 Pain: 1 Burning: 1 Itching: 67	Prodromal phase: 3 Active phase: 43 Convalescence phase: 25	Resolved few days later (mean: 12.7 days)	N/M	49 cases were confirmed by PCR. Others were not tested.
Chilblain	1 [22]	27	F	Kuwait	Dorsal aspect of fingers	Asymptomatic	Asymptomatic for COVID-19	N/M	No medication/no exposure to cold	PCR
	1 [22]	35	F	Kuwait	Dorsal aspect of fingers	Asymptomatic	Asymptomatic for COVID-19	N/M	No medication/no exposure to cold	PCR
	3 [13]	14-22	N/M	France	Toes of both feet	Pain and burning	Asymptomatic for COVID-19	N/M	No medication/no exposure to cold	Not tested
	1 [15]	28	F	Spain	Both heels	Itching	Active phase	N/M	No medication/no exposure to cold	N/M
	1 [23]	26	M	Italy	Both heels	Asymptomatic	Asymptomatic for COVID-19	N/M	No medication/no exposure to cold	Not tested
	1 [23]	16	F	Italy	Both heels	Asymptomatic	Asymptomatic for COVID-19 except for pharyngodynia 2 weeks ago	N/M	No medication/no exposure to cold	Not tested
	1 [23]	18	F	Italy	Both heels & extensor surfaces of toes	Asymptomatic	Asymptomatic for COVID-19	N/M	No medication/no exposure to cold	Not tested
	1 [23]	48	M	Italy	Extensor surfaces of both heels	N/M	Active phase	N/M	No medication/no exposure to cold	Not tested
Vesicular rash	22 [24]	Median age = 60	M (16 cases) F (6 cases)	Italy	All had truncal lesions ± involvement of extremities	Mild itching in 9 cases	Active phase	Resolved 4-15 days later	No medication	All cases were confirmed by PCR.
	1 [6]	N/M	N/M	Italy	Mainly trunk	N/M	Active phase	Resolved few days later	N/M	PCR
	1 [25]	8	F	Italy	Trunk	Asymptomatic	Active phase	Resolved 7 days later without any treatment	No medication	PCR
	2 [7]	N/M	N/M	Italy	Trunk (localized herpetiform)	Mild itching	Active phase	N/M	N/M	N/M
	1 [7]	N/M	N/M	Spain	Back (localized herpetiform)	N/M	Active phase	N/M	N/M	N/M
	34 [15]	Mean: 45.6	F: 19 M: 15	Spain	N/M	Asymptomatic: 6 Pain: 3 Burning: 2 Itching: 23	Prodromal phase: 5 Active phase: 19 Convalescence phase: 10	Resolved few days later (mean: 9.3 days)	N/M	17 cases were confirmed by PCR. Others were not tested.
Livedo reticularis	1 [25]	67	M	USA	Anterior thigh	Asymptomatic	Active phase	Resolved 19 hours later without any treatment	N/M	PCR
	1 [25]	47	F	USA	Right lower limb	Asymptomatic	Convalescence phase	Resolved 20 minutes later without any treatment	No medication but appeared after sun exposure	PCR
	21 [25]	Mean age: 63.1	F: 10 M: 11	Spain	N/M	Asymptomatic: 15 Pain: 1 Burning: 2 Itching: 3	Prodromal phase: 1 Active phase: 18 Convalescence phase: 2	Resolved few days later (mean: 9.4 days)	N/M	17 cases were confirmed by PCR. Others were not tested.
Petechiae	1 [26]	N/M	N/M	Thailand	N/M	N/M	Prodromal phase	N/M	N/M	PCR
	1 [14]	48	M	Spain	Buttocks, popliteal fossa, proximal anterior thighs, lower abdomen	Itching	Active phase	Resolved 5 days later with 0.05% betamethasone cream+loratadine	No new medication	PCR

M: male; F: female; N/M: not mentioned; PCR: polymerase chain reaction; ICU: intensive care unit.

**Table 2:** Classification and characteristics of the cutaneous lesions reported in association with COVID-19.

Cutaneous manifestation (total number of reported cases)	Number of patients	Age, mean (range) (years)	Sex	Country	Location of skin lesions	Dermatologic symptoms	Phase of COVID-19 in which skin lesions appeared	Method of COVID-19 diagnosis
Maculopapular rash	200	60.6 (6-89)	F: 102 M: 82 N/M: 16	Spain: 176 Italy: 17 France: 4 USA: 2 Thailand: 1	Trunk: 14 Generalized (erythroderma): 8 Face & upper body: 2 N/M: 176	Asymptomatic: 64 Itching: 104 Burning: 10 Pain: 4 N/M: 18	Prodromal: 9 Active: 131 Convalescence: 60	146 cases were confirmed by PCR. Others were not tested or N/M.

Urticaria	84	47.6 (2 months–71 years) N/M: 5	F: 52 M: 27 N/M: 5	Spain: 74 Italy: 4 France: 3 Belgium: 2 Thailand: 1	Trunk: 3 Generalized: 4 Face & upper body: 3 N/M: 73	Asymptomatic: 9 Itching: 73 Burning: 1 Pain: 1	Prodromal: 5 Active: 52 Convalescence: 25 N/M: 2	57 cases were confirmed by PCR. Others were not tested or N/M.

Chilblain	81	31.76 (14-48)	F: 53 M: 25 N/M: 3	Spain: 72 Italy: 4 France: 3 Kuwait: 2	Fingers: 2 Heel & toes: 79	Asymptomatic: 24 Itching: 22 Burning: 11 Pain: 26	Prodromal: 5 Active: 26 Convalescence: 42 Asymptomatic carrier of COVID-19: 8	31 cases were confirmed by PCR. Others were not tested or N/M.

Vesicular rash	61	Not reportable	F: 26 M: 31 N/M: 4	Spain: 26 Italy: 35	Trunk: 26 (2 of them localized & herpetiform) N/M: 35	Asymptomatic: 20 Itching: 34 Burning: 2 Pain: 3 N/M: 2	Prodromal: 5 Active: 46 Convalescence: 10	41 cases were confirmed by PCR. Others were not tested or N/M.

Livedo reticularis	23	62.5	F: 11 M: 12	Spain: 21 USA: 2	Lower limb: 2 (unilateral) N/M: 21	Asymptomatic: 17 Itching: 3 Burning: 2 Pain: 1	Prodromal: 1 Active: 19 Convalescence: 3	19 cases were confirmed by PCR. Others were not tested or N/M.

Petechiae	2	1 patient: 48 1 patient: N/M	M: 1 N/M: 1	Spain: 1 Thailand: 1	Lower half of the body: 1 N/M: 1	N/M Itching	Prodromal: 1 Active: 1	Both cases were confirmed by PCR.

M: male; F: female; N/M: not mentioned; PCR: polymerase chain reaction.
